# Supplementary material for: Isolation of Bacillus altitudinis 5-DSW with Protease Activity from Deep-Sea Mineral Water and Preparation of Functional Active Peptide Fractions from Chia Seeds
Source: Microorganisms. 2024 Oct 10;12(10):2048. doi: 10.3390/microorganisms12102048 (PMC11509774; doi:10.3390/microorganisms12102048)
Supplement: Supplementary file 1 [file microorganisms-12-02048-s001.zip › microorganisms-3189450-supplementary.pdf]

Supplementary Materials:

Supplement Table S1. Sugar utilization profile of *Bacillus* strains

Carbohydrate	Characteristic reaction in strain:				Carbohydrate	Characteristic reaction in strain:			
	1	5	8	11		1	5	8	11
**0**	-	-	-	-	**ESC**	+	+	+	+
**GLY**	+	+	+	+	**SAL**	+	+	+	+
**ERY**	-	+	-	-	**CEL**	+	+	+	+
**DARA**	-	+	-	-	**MAL**	-	+	-	+
**LARA**	+	+	+	+	**LAC**	-	+	-	-
**ROB**	+	+	+	+	**MEL**	-	+	+	-
**DXYL**	+	+	+	+	**SAC**	+	+	+	+
**LXYL**	-	+	-	-	**TRE**	+	+	+	+
**ADO**	-	+	-	-	**INU**	-	-	-	-
**MDX**	-	+	-	-	**MLZ**	-	-	-	-
**GAL**	+	+	+	+	**RAF**	-	+	+	-
**GLU**	+	+	+	+	**AMD**	-	+	-	+
**FRU**	+	+	+	+	**GLYG**	-	+	-	+
**MNE**	+	+	+	+	**XLT**	-	-	-	-
**SBE**	-	+	-	-	**GEN**	+	+	+	+
**RHA**	-	+	-	+	**TUR**	-	-	-	-
**DUL**	-	+	-	-	**LYX**	-	-	-	-
**INO**	-	+	-	+	**TAG**	+	-	+	-
**MAN**	+	+	+	+	**DFUC**	-	-	-	-
**SOR**	-	+	-	+	**LFUC**	-	-	-	-
**MDM**	+	-	+	-	**DARL**	-	-	-	-
**MDG**	-	+	-	+	**LARL**	-	+	-	-
**NAG**	+	+	+	+	**GNT**	-	+	+	-
**AMY**	+	+	+	+	**2KG**	-	-	-	-
**ARB**	+	+	+	+	**5KG**	-	+	-	-

Data obtained by API 50CHB kit, '+': positive, '-': negative

0=Control; GLY=Glycerol; ERY=Erythritol; DARA=D-Arabinose; LARA=L-Arabinose; ROB=Ribose; DXYL=D-xylose; LXYL=L-xylose; ADO=Adonitol; MDX=Methyl-b-d-xylopyranside; GAL=Galactose; GLU=Glucose; FRU=Fructose; MNE=Mannose; RHA=Rhamnose; DUL=Dulcitol; INO=Inositol; MAN=Mannitol; SOR=Sorbitol; MDM=Methyl-α; D-mannopyranside; MDG=Methyl-α, D-glucoside; NAG=N-Acetyl-glucosamine; AMY=Amygdaline; ARB=Arbutine; ESC=Esculin; SAL=Salicin; CEL=Cellobiose; MAL=Maltose; LAC=Lactose; MEL=Melibiose; SAC=Sucrose; TRE=Trehalose; INU=Inulin; MLZ=Melezitose; RAF=Raffinose; AMD=Starch; GLYG=Glycogen; XLT=Xylitol; GEN=Gentiobiose; TUR=D-Turanose; LYX=D-Lyxose; TAG=D-Tagatose; DFUC=D-Fucose; LFUC=L-Fucose; DARL=D-Arabitol; LARL=L-Arabitol; GNT=Gluconate; 2KG=2-keto-Gluconate; 5KG=5-keto-Gluconate

Supplement Table S2. Identification of bacterial strains by 16S rDNA sequencing

**Sample**	**Closest relative**	**Sequence homology (%)**
**1**	*Bacillus pumilus* strain L17 (KC778622)	100%
	Bacillus altitudinis strain SH164 (KC172059)	100%
	Bacillus stratosphericus strain SH130 (KC172047)	100%
	Bacillus pumilus strain GC43 (KF158227)	100%
	Bacillus aerophilus strain SH31 (KC172027)	100%
	Bacillus subtilis strain YNA61 (JQ039972)	100%
**5**	*Bacillus altitudinis* strain EkA2-2 (KF017559)	100%
	Bacillus pumilus strain ML484 (KC692175)	100%
	Bacillus subtilis strain D35 (KC441771)	100%
	Bacillus stratosphericus strain L67 (KC934796)	100%
	Bacillus aryabhattai strain N08 15 (JX988388)	100%
	Bacillus safensis strain 3-5 (JX867749)	100%
**8**	*Bacillus stratosphericus* strain H76 (KC934845)	100%
	Bacillus pumilus strain GR35 (KC771050)	100%
	Bacillus aerophilus strain 20E (KC329821)	100%
	Bacillus pumilus strain MB4 NIOT (HQ858060)	100%
	Bacillus altitudinis strain IHB B 1045 (KF475828)	99%
	Bacillus pumilus strain ESR21 (KC915229)	99%
**11**	*Bacillus flexus* strain S858 (KC523392)	100%
	Bacillus megaterium strain S857 (KC523391)	100%
	Bacillus aryabhattai strain M54 (KC934883)	100%
	Bacillus megaterium strain IHB B 4625 (KF475802)	100%
	Bacillus horikoshii strain IARI-HHS2-13 (KF054756)	100%
	Bacillus aryabhattai strain IHB B 4622 (KF475797)	100%

Supplement Table S3. Primers used for 16S rDNA, *recA*, and *atpD* gene amplification

Target for amplification	Primer name	Oligonucleotide (5′–3′)	PCR cycling conditions	References
16S rDNA(20~22bp)	27F	AGAGTTTGATCNTGGCTCAG		This study
	1492R	TACGGYTACCTTGTTACGACTT		
recA (540 bp)	*recA*-F	GATCGTCAAGCAGCCTTAGAT	5 min 95 °C, 30*(30 s 95 °C, 30 s 45 °C, 1 min 72 °C), 5 min 72 °C	[53]
	recA-R	TTACCGACCATAACGCCGAC		
atpD (519 bp)	*atpD*-F	CAAGTCATGGGTCCGGTTGT	5 min 95 °C, 30*(30 s 95 °C, 30 s 45 °C, 1 min 72 °C), 5 min 72 °C	[54]
	atpD-R	CGCCGTGCTCTTGGGCGATGTT		
